# Applications and Tuning Strategies for Transcription Factor-Based Metabolite Biosensors

**DOI:** 10.3390/bios13040428

**Published:** 2023-03-28

**Authors:** Gloria J. Zhou, Fuzhong Zhang

**Affiliations:** 1 Department of Energy, Environmental and Chemical Engineering, Washington University in St. Louis, St. Louis, MO 63130, USA; gjzhou@wustl.edu; 2 Division of Biology & Biomedical Sciences, Washington University in St. Louis, St. Louis, MO 63130, USA; 3 Institute of Materials Science & Engineering, Washington University in St. Louis, St. Louis, MO 63130, USA

**Keywords:** transcription factor, biosensor tuning, biosensor applications, transcriptional control, translational control, high-throughput screening, dynamic regulation, metabolic heterogeneity

## Abstract

Transcription factor (TF)-based biosensors are widely used for the detection of metabolites and the regulation of cellular pathways in response to metabolites. Several challenges hinder the direct application of TF-based sensors to new hosts or metabolic pathways, which often requires extensive tuning to achieve the optimal performance. These tuning strategies can involve transcriptional or translational control depending on the parameter of interest. In this review, we highlight recent strategies for engineering TF-based biosensors to obtain the desired performance and discuss additional design considerations that may influence a biosensor’s performance. We also examine applications of these sensors and suggest important areas for further work to continue the advancement of small-molecule biosensors.

## 1. Introduction

Small-molecule biosensors play an important role in the real-time monitoring of metabolite concentrations during microbial fermentations. The ability to sense and respond to intracellular metabolites is crucial for better understanding and regulating pathway behavior for enhanced metabolite production [1]. Many different types of biosensors have been developed and characterized in microbial hosts, including aptamers, riboswitches, fluorescence resonance energy transfer (FRET)-based sensors, and transcription factor (TF)-based sensors [2,3,4,5]. Aptamers are nucleic-acid-based biosensors that undergo a conformational change upon the binding of a target metabolite. These biosensors can be used for the detection of harmful toxins, such as mycotoxins from fungi [6] or microcystins from cyanobacteria [7]. A more detailed examination of aptamer-based small-molecule biosensors can be found in a review by Pfeiffer and Mayer [8]. Another type of nucleic-acid-based biosensor is the riboswitch, which regulates gene expression using structures in the 5′ untranslated region of mRNA to recognize small molecule inducers [9]. In contrast, FRET sensors are engineered proteins containing a sensing domain and a pair of fluorescent proteins, which undergo fluorescence resonance energy transfer upon binding of the target metabolite. FRET sensors have previously been used to study in vivo ATP dynamics [10,11] and are reviewed elsewhere [12,13]. Some TFs change their DNA-binding activities after interacting with their target metabolites. These TFs can be engineered as sensors to report metabolite concentrations by fusing a reporter gene to TF-responsive promoters and measuring the associated output signals. One advantage of TF-based sensors is their modularity, which makes their performance tunable for various engineering applications. While previous reviews on this topic often summarize TF-based sensors for different metabolites [14,15], this review will focus on tuning strategies for TF-based biosensors that can be used for applications in different microbial hosts or conditions. Biosensors assisted by photonics, plasmonics, or nanoscale materials are beyond the scope of this review and are described in detail elsewhere [16,17,18].

Depending on the number of components involved, TF-based sensors can be categorized as one-component sensors, where the sensor and regulator domains are encoded by one protein, or two-component sensors, where the two domains are encoded separately [19,20]. Two-component sensors often respond to extracellular signals, whereby an input signal causes a conformational change in the histidine kinase sensor, leading to the phosphorylation of the kinase enzyme which then binds the response regulator to carry out downstream processes [21]. An example of a two-component signaling system is the aspartate-responsive Taz-OmpR system in *Escherichia coli*, which was engineered to detect aspartate over a wide concentration range (from 4 to 67 µM) [21]. Two-component biosensors have been reviewed previously [19]. This review will specifically focus on one-component biosensors, which are often used to sense intracellular metabolites. Furthermore, this review will specifically examine sensors that are used in prokaryotic cells. Research and reviews on sensors for eukaryotic cells can be found elsewhere [22,23,24,25,26].

**Figure 1:**
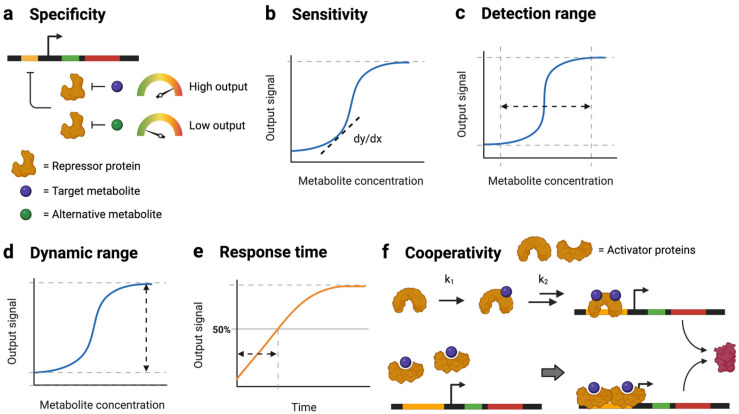
Biosensor performance criteria. (**a**) Specificity of a biosensor for the target metabolite compared to alternative metabolites; (**b**) sensitivity of the biosensor as represented by the slope of the sensor’s response curve; (**c**) detection range reflects the limits of the metabolite concentration detected by a sensor; (**d**) dynamic range reflects the ratio of maximal output over minimal output levels; (**e**) response time quantifies the time it takes for a sensor to reach half of its steady-state signal; (**f**) mechanisms affecting biosensor cooperativity.

Several different criteria are used to evaluate biosensor performance, including specificity, sensitivity, detection range, dynamic range, response time, and cooperativity (Figure 1). The specificity of a sensor refers to the difference in output signal intensity upon the binding of the target ligand relative to alternative ligands (Figure 1a) [27]. High specificity reduces the likelihood of false positives to ensure that the biosensor output is an accurate reflection of the target metabolite’s concentration. Sensitivity measures the amount of change in a biosensor’s output signal when the concentration of its detected metabolite changes (Figure 1b). Greater sensitivity can provide more detailed information related to subtle changes in a metabolite’s concentration [28]. Detection range refers to the range between the upper and the lower limits of a metabolite’s concentration that a biosensor can respond to (Figure 1c). If the metabolite levels are above or below the detection range, then the associated biosensor would not be able to provide any useful information. Dynamic range refers to the highest fold-change in the biosensor’s output levels compared to the lowest uninduced level (Figure 1d). A greater dynamic range can provide more information on the dynamic properties of the metabolite that is being measured and is especially important for avoiding signal saturation [29]. The response time considers how quickly an output signal can reach its half-maximal value following the induction of the biosensor (Figure 1e) [30]. Faster response times are particularly important for targeting toxic compounds [31]. Lastly, cooperativity describes the shape of a sigmoidal dose-response curve. High cooperativity can arise from different biological mechanisms: (1) the binding of the first metabolite makes it easier for additional metabolite molecules to bind [32]; and (2) TFs bind to a target DNA as multimers, such as the QacR dimer in *Staphylococcus aureus* (Figure 1f) [33]. Many of these behaviors can be quantified using the dose-response curves of a biosensor. Tuning these properties is essential to adopting any biosensor for specific applications.

Rapid advances in synthetic biology research necessitate efficient strategies for biosensor development. The goal of this review is to examine tuning strategies from the perspective of typical cellular operations and consider how biosensors might be used for different applications in research and the industry. We aim to equip readers with strategies for adopting published sensors for user-specific applications. First, we will evaluate tuning strategies that have led to recent advances in biosensor construction. These strategies can be divided into three categories based on the sensing component, including promoter engineering, TF engineering (transcriptional control), and ribosome binding site (RBS) engineering (translational control). The latter half of this review is dedicated to three important applications of metabolite biosensors: high-throughput screening, visualizing metabolite dynamics and performing dynamic metabolic regulation, and investigating metabolic heterogeneity. High-throughput screening and dynamic regulation are well-established applications of TF-based biosensors with key roles in biosensor discovery and metabolic control [15,34]. We will further highlight the use of TF-based sensors for the study and control of metabolic heterogeneity, which has critical implications in our understanding of single-cell behavior and large-scale bioproduction. Lastly, we will provide our conclusions and outlook for the future development of small-molecule biosensors.

## 2. Tuning Strategies

**Figure 2:**
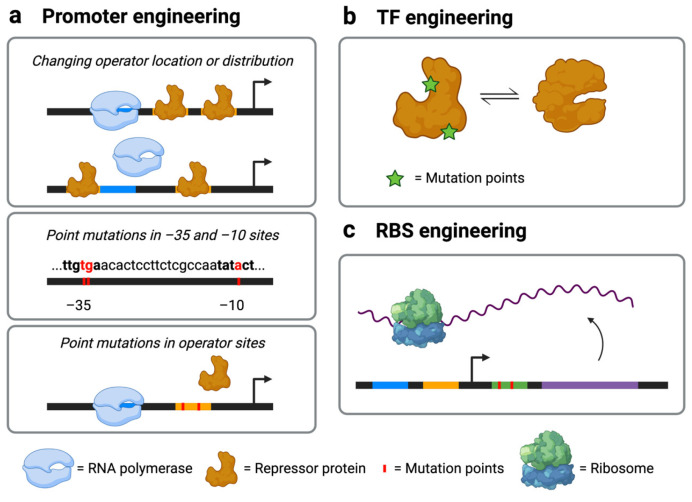
Tuning strategies for biosensors. (**a**) Promoter engineering through changing the number and location of operator sites, mutating the −35 or −10 region of the promoter, or mutating the transcription factor (TF)-binding operator sites (the black bar represents the DNA sequence; the blue segment represents the RNA polymerase binding site; the orange segment represents the TF-binding site; additional icons are defined in the Figure); (**b**) TF engineering via mutations to the ligand-binding pocket or the DNA-binding domain; (**c**) ribosome-binding site (RBS) engineering (the green segment represents the RBS sequence; the purple segment represents the gene of interest; additional icons are defined in the Figure).

Biosensors that have been optimized in one lab condition may perform poorly under a different condition, such as when growing cells using a different carbon source or in a large-scale fermenter. When designing or tuning a sensor, a good practice is to select sensor components (including metabolite-responsive TFs, promoters, and other genetic elements such as the RBS) that are orthogonal to the host system to avoid cross-talk [35]. In this section, we will discuss tuning strategies targeted to each biosensor component and performance criteria (Figure 2). The advantages and disadvantages of each strategy are summarized in Table 1.

### 2.1. Engineering TFs for Optimal Sensor Performance

As the central sensing component, the ligand-responsive TF has multiple biochemical characteristics which can deeply affect a sensor’s performance, including the TF expression level, its binding affinity for the ligand, and its binding affinity for the target DNA. For many TF-based sensors, tuning the expression level of the TF is an important step [41]. TF expression at levels that are too low is insufficient to change a reporter’s expression, resulting in low sensitivity and a small dynamic range; in contrast, TF expression at levels that are too high permanently turns the reporter on (if the TF is an activator) or off (if the TF is a repressor) [42]. The most optimal TF expression level changes with the copy number of its operators in the sensor strain. The actual TF expression level also changes with cell growth conditions, which will need to be accounted for when using a sensor in different settings [43]. Increasing the copy number of TFs or reporter proteins can be used to create serial or parallel circuits, which have been shown capable of improving sensor sensitivity by 9-fold with minimal leaky expression and enhancing output signal intensity by 3.65-fold, respectively [44]. Additionally, the affinities of a TF for its ligand and DNA operator are important for the sensor’s performance. These two parameters influence a sensor’s basal signals, threshold, and dynamic range. For sensors with a repressed-repressor architecture, increasing the binding affinity between a TF and its operator decreases the basal level of the sensor while increasing the detection threshold and sensor dynamic range [40]. The exact relationships between these sensor parameters and sensor performance metrics have previously been described by a phenomenological model [40]. These binding affinities can be tuned by mutating the ligand-binding site of the TF or by mutating the operator sequence (Figure 2a). These strategies are particularly effective for optimizing a sensor’s dynamic range [40].Additionally, TFs can be engineered to improve their specificity for target metabolites [45]. For example, substitutions in the TF ligand-binding domain increased CamR responsivity to different terpenes including camphor, borneol, fenchol, eucalyptol, and camphene [36]. Similarly, Gong et al. introduced mutations in the ligand-binding pocket of TrpR, which created variants with up to three-fold higher specificity for tryptophan over 5-hydroxytryptophan [54]. TFs can be further engineered to sense alternative metabolites (Figure 2b). This process typically involves screening and selecting for desirable TF mutants. For example, LuxR was artificially evolved in this manner to no longer respond to 3-oxo-hexanoyl-homoserine lactones while retaining the ability to recognize straight-chain acyl-homoserine lactones [55]. Similarly, Wu et al. engineered BmoR to differentiate between constitutional isomers of different alcohol molecules. The resulting BmoR variants demonstrated specific responses to either *n*-butanol or isobutanol [56]. In some cases, it may be necessary to construct a new biosensor by piecing together smaller parts. Rogers and Church developed a PrpR-based biosensor that responds to 3-hydroxyproprionate using helper enzymes that convert the target metabolite into 2-methylcitrate, which can be sensed by PrpR [57]. For a detailed discussion of how to engineer TFs to sense different ligands, please refer to another review in this Special Issue.

### 2.2. Engineering Promoters for Optimal Sensor Performance

TF-responsive promoters transcriptionally control the rate of a biosensor’s output and can also be engineered to tune a sensor’s performance (Figure 2a). Promoter engineering is useful for fine-tuning a sensor’s sensitivity, detection range, dynamic range, or signal output intensity. The TF-responsive promoter often contains one or two operator sites where the ligand-responsive TF binds. Changing the number or location of the operator sites affects the output intensity, cooperativity, and dynamic range. d’Oelsnitz et al. constructed a biosensor containing two CamR operator sequences with greater symmetry than the native operator, which increased the dynamic range by more than two-fold [36]. The introduction of additional repressor operator sites is often effective for reducing leaky gene expression, thereby also improving the dynamic range. Chen et al. introduced two additional LacO_1_ and TetO_2_ sites to their biosensor system on either side of the +1 site. The multiple operator sites contributed to dynamic ranges spanning four orders of magnitude [37].

Furthermore, point mutations at different positions within the promoter can be used to tune the output signal levels [38]. Xiao et al. achieved a 14-fold improvement in the detection range of D,L-lactate through mutations in the LldR operator site [39]. Mutations introduced to the LacI operator site by Mannan et al. changed the basal signal levels by two orders of magnitude and changed the maximal output levels up to two-fold [40]. Meanwhile, mutations in the −35 and −10 sites affect the binding between RNA polymerase (RNAP) and the promoter, controlling the transcription initiation rate of the reporter protein. Xiao et al. introduced two degenerate nucleotides into the −10 box of a constitutive promoter to construct a synthetic promoter library controlling the production of GlnA and GlnR TFs. The resulting mutants achieved two- to three-fold differences in the repression of the fluorescent reporter protein [41]. d’Oelsnitz et al. introduced their selected changes into the −10 region of a CamR-repressible promoter and showed that a single G-to-A base substitution could enhance the biosensor response to camphor by almost four-fold [36]. This approach applies not only to repressor-based architectures but also to activator-regulated promoters. Chen et al. made base modifications in the consensus −10 and −35 sites of AraC- and LasR-regulated promoters, which changed the transcription rates by four orders of magnitude [37]. It is worth noting that modifying the affinity between RNAP and a TF-responsive promoter can also result in large changes in the sensor output, which may not be ideal for certain applications.

### 2.3. RBS Engineering (Translational Control)

RBS engineering (Figure 2c) provides a method of translational control over biosensor performance. Like the −35 and −10 sites in the promoter region, the RBS site is an essential feature of every genetic construct and can be engineered to manipulate the translation initiation rate of either the TF or the reporter protein. Ding et al. constructed a library of cross-RBSs, which combined the RBS sequences for CdaR and sfGFP and enhanced the dynamic range of a glucarate-sensitive biosensor up to three orders of magnitude [49]. Similarly, the translation initiation region can be synthetically evolved to increase the dynamic range. Shilling et al. introduced mutations to the translation initiation region of an arabinose-inducible promoter, increasing the maximum reporter levels by five times to improve the dynamic range with minimal effects on the sensitivity and detection range [50]. Different RNAP can also be incorporated into a regulation module to construct novel gene circuit architectures. Wang et al. constructed a genetic AND gate using a σ^54^-dependent *hrpR*/*hrpS* regulation module instead of σ^70^-based control and achieved a near-digital logic behavior that was modular and orthogonal to the host system [51]. Translational control can be further combined with transcriptional control in multi-layer control strategies to reduce leaky gene expression and improve the dynamic range. Greco et al. constructed and compared three multi-level controllers with the baseline performance of an IPTG-inducible P_*tac*_ promoter. Among the different designs, the addition of a toehold switch sequence to the 5′ end of a reporter protein provided the most desirable performance characteristics. This second layer of control increased the dynamic range by two orders of magnitude with minimal changes to the cell doubling and lag times when compared to the single-layer P_*tac*_ architecture [52].

### 2.4. Additional Design Considerations

#### 2.4.1. Growth-Dependent Sensor Performance

All sensor engineering efforts should consider cell growth, available resources, and the metabolism of the microbial host. Microbial growth rates affect the concentration of many sensor components, so sensor performance is unavoidably affected by cell growth. When the growth rate changes, such as when cells are switched from a well-controlled lab environment to industrially relevant conditions, the sensor’s dynamic range and signal levels may change drastically due to changes in the cell growth rate. Using three TF-promoter systems with repressed-repressor architectures, including TetR-P_*tet*_, LacI-P_lacUV5_, and FadR-P_AR_, Hartline et al. found that the dynamic ranges of the aTc and IPTG sensors increased with an increasing growth rate, while the dynamic range of the fatty acid (FA) sensor decreased with an increasing growth rate [43]. Further modeling work suggested that differences in growth rate dependence are caused by different metabolite transport mechanisms. Cell growth dilutes the concentration of transporter proteins, which affects the intracellular concentration of extracellular inducers differently depending on whether they are actively imported or exported [43]. Additionally, competition between the reporter protein (e.g., GFP) and native proteins for available resources, such as free ribosomes, may also affect a sensor’s performance, particularly when cells are grown in a nutrient-poor medium [58,59].

#### 2.4.2. Tradeoffs between Sensor Performance Parameters

There are also tradeoffs related to the output signal to consider when designing a biosensor. The coupling of sensor performance parameters introduces additional challenges to the development of an ideal biosensor. For example, increasing the maximal sensor output levels by using a strong RBS can simultaneously increase the basal signal [40]. A previous mathematical analysis revealed that increasing a sensor’s dynamic range also increases its response threshold [40]. Additionally, evolving a TF to sense new metabolites may reduce the sensor’s specificity [36]. The decision of which feature to prioritize depends on the application’s needs. High specificity is important if the biosensor is being used to differentiate a target metabolite from other coexisting compounds, such as when detecting bile salts as a biomarker for several different liver diseases [60]. On the other hand, a higher detection limit might be advantageous in cases where the studied system is expected to generate a high signal noise [61].

## 3. Applications

**Figure 3:**
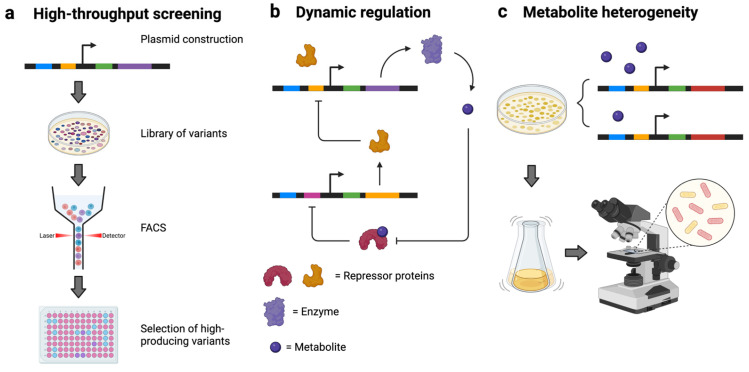
Applications of TF-based metabolite biosensors. (**a**) High-throughput screening of genetic libraries to select active enzymes or high-producing strains; (**b**) metabolite sensor-enabled dynamic regulation of metabolic pathways (example of a negative feedback loop to control metabolite production); (**c**) studying metabolite heterogeneity within an isogenic cell population using metabolite biosensors.

Metabolite biosensors have multiple biotechnological applications. Reviews on their applications in environmental detection and diagnostics can be found elsewhere [62,63]. This Special Issue also contains a detailed review of biosensors for natural products. Here, we will focus on the use of biosensors for metabolic engineering purposes (Figure 3).

### 3.1. High-Throughput Screening

Metabolite biosensors can be used with fluorescence-activated cell sorting (FACS) for the high-throughput screening of engineered enzymes or microbial strains by measuring the output signals of a fluorescent reporter gene fused to a TF-responsive promoter (Figure 3a). Biosensors that detect the product concentration of an enzymatic reaction can be used to screen active enzymes from different organisms [64] or from a mutagenic library [65]. Sensors that detect the product of a metabolic pathway have previously been used to optimize pathway enzyme expression and flux [66,67]. Additionally, sensors can be used for the high-throughput screening of sensor variants [48,68]. Li et al. created a library of phthalic/terephthalic acid sensors by mutating the phthalic/terephthalic acid-responsive TF XylS. Using FACS, sensors that can detect as low as 10 µM phthalic/terephthalic acid were identified, representing a 2.2- and 3.0-fold improvement in the sensor’s sensitivity compared to that achieved with the wild-type XylS. These sensors were further used to screen for phthalate ester hydrolase, leading to the identification of new enzyme variants with 2- and 2.5-fold enhancements in the degradation of dibutyl phthalate and *p*-nitrophenyl butyrate, respectively [69]. Screening can also be used to characterize a promoter’s behavior in response to cell wall biosynthesis inhibitors, expanding the repertoire of available tools for antimicrobial treatments [70]. As strategies for creating and screening large libraries further mature, metabolite biosensors will continue to be used in high-throughput screening for various applications.

### 3.2. Metabolite Dynamics and Dynamic Metabolic Regulation

In microbial strains, intracellular metabolite concentrations often vary throughout the course of bioproduction, and each engineered pathway enzyme must be expressed at the right time and in the right amount to properly adjust the dynamic metabolism and prevent metabolic intermediates from accumulating to toxic levels [71]. TF-based biosensors are useful tools for visualizing metabolite dynamics (i.e., the dynamic concentration change of a metabolite) in living cells [11,72], which is a challenging task using conventional analytical methods. Furthermore, TF-based sensors can provide real-time adjustments of pathway enzyme expression in response to changing metabolite concentrations, thus providing dynamic control to engineered metabolic pathways (Figure 3b). Dynamic metabolic control is more advantageous than static control and is very effective for improving a product’s titers and yields [73,74]. For example, Zhou et al. engineered a sensor that responds to both (2*S*)-naringenin and *p*-coumaric acid and used this sensor to control the synthesis and consumption of malonyl-CoA. This multi-layer dynamic regulation network improved naringenin production by 8.7-fold and increased cell growth by 20% [75]. Essential processes within the system of the selected host strain can serve as the basis for synergistic strategies to enhance the product yield without compromising cell robustness. A comprehensive review of dynamic metabolic control can be found elsewhere [76].

When metabolite biosensors are used to create dynamic metabolic controls, the sensors need to be tuned to meet the specific regulation requirements, such as having a proper metabolite detection range that matches the accumulated intermediate concentration [77] or displaying a proper output range that fits the desired expression level of under-controlled enzymes [42]. Furthermore, a sensor can be used to regulate pathway flux in different ways depending on which enzymes are controlled and how they are controlled, thus leading to different control topologies (e.g., negative feedback, positive feedforward, etc.). Different control topologies have different response times and can result in drastically different product titers and yields. Liu et al. found that negative metabolic loops rapidly stabilize the concentration of the metabolite that the sensors detect and control, but a multi-layered negative metabolic loop can easily create a metabolic overshoot that may be undesirable for metabolite bioproduction applications [78]. The experimental testing of sensors with different dynamic control topologies can be very time-consuming. Fortunately, recent progress in computational modeling using multi-objective optimization can help to identify the most optimal design strategy [79]. Verma et al. used this approach and tested tradeoffs in growth and production among three different negative feedback circuits, demonstrating the optimality of a genetic architecture that improved glucaric acid production by 2.5-fold [79].

Sensor-enabled dynamic regulation has been found to be useful not only in metabolic engineering but also in healthcare. Koh et al. designed a sensor-actuator system to reduce *Clostridium difficile* infections by detecting sialic acid and converting conjugated bile salts into unconjugated forms. Murine models showed a 100% survival rate in the treated group compared to a 14.3% survival rate in the no-sensor control group, and the average Clinical Sickness Score for the treated group was at least half that of the untreated control group [80]. An important note to make is that these biosensors rely on the detection of pathway-specific metabolites and are limited in the scope of their application. Additionally, the lack of a reporter to track the enzyme behavior can potentially introduce challenges during strain characterization. The use of fluorescent protein tags is recommended for monitoring the dynamics of a gene of interest over time with time-lapse fluorescence microscopy [81].

### 3.3. Illuminating and Engineering Metabolic Heterogeneity

Fluorescence microscopy can be further coupled with TF-based biosensors to study cell-to-cell variations in metabolite levels among an isogenic cell population, which affect both metabolite-specific functions and general cellular processes such as cell growth [82,83,84]. TF-based biosensors offer a platform for measuring metabolite concentrations in single cells, which is difficult to quantify using conventional analytical tools (Figure 3c). For example, Mustafi et al. constructed a TF-based sensor for measuring single-cell variations in L-valine production in *Corynebacterium glutamicum* using EYFP fluorescence. The authors were able to track a cell lineage using a microfluidic device and observed that the final 38 single cells had significant differences in their cell size, growth rate, and production levels [85]. Similarly, Xiao et al. studied an *E. coli* strain engineered to overproduce FAs and revealed that 15% of the cell population was responsible for more than half of the overall FA titer [86]. These studies have shown the prevalence of metabolic heterogeneity at the single-cell level, which highlights the importance of developing biological tools for better understanding and regulating this phenomenon. When studying variations at the single-cell level, it is important to keep in mind the source of the variations and how experimental or equipment parameters might influence the observed values, such as with the signal-to-noise ratios of fluorescence microscopes [87,88].

Metabolic heterogeneity can be further exploited to improve the overall bioproduction titers and yields using TF-based metabolite sensors. Xiao et al. developed a method called PopQC which involves using a metabolite product biosensor to increase the number of high-producing cells [86]. In this design, high intracellular FA levels trigger an FA sensor to express a selection gene, which confers a growth advantage on high-producing cells in the presence of the selection pressure, subsequently improving the overall FA titers and productivity by threefold [86]. Similarly, Rugbjerg et al. replaced native essential gene operon promoters in *E. coli* with a mevalonic acid-responsive biosensor. This strategy enabled the mevalonic acid production levels to be maintained at a steady state for over 50 generations after non-engineered cells had already ceased its production [89]. Therefore, TF-based biosensors are useful for both studying and controlling metabolic heterogeneity.

## 4. Conclusions and Outlook

**Figure 4:**
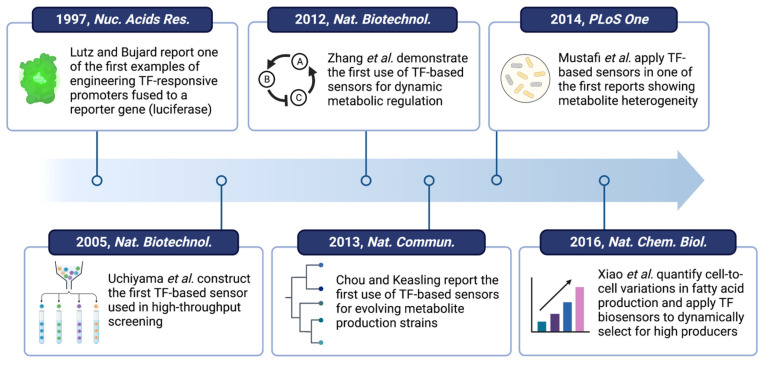
Roadmap of notable work related to the development and application of TF-based metabolite biosensors [77,86,94,95,96,97].

TF-based metabolite biosensors continue to remain an essential tool for advancing research and industrial bioproduction (Figure 4) [67,90,91]. The tunability of these biosensors allows them to be easily applied in various genetic architectures for producing valuable small molecules [92]. However, these biosensors may have both direct and indirect effects on the host metabolism, such as the burden caused by competition for cellular resources [93] or the toxicity caused by TF overexpression [58,79], and may not work well when transferred from one microbial strain to another. These challenges can be overcome by tuning the sensor components as discussed above. At the same time, it is important to consider other design variables, such as the growth medium and tradeoffs between sensitivity and background noise, to develop a fully optimized system in practice.

There have been significant advances in the number of TFs discovered that can sense and respond to various small molecules [98], opening the doors for new capabilities and exciting applications. First, TF-based sensors that respond to different small molecules can be integrated into gene circuits to simultaneously sense multiple chemicals, allowing engineered microbes to make decisions based on complex metabolic or chemical conditions [99]. Second, machine learning models have proven to be powerful tools for studying many complex biological processes [100,101,102] and can be useful for dissecting a sensor’s parameters and properties to create systems with more precise and enhanced performance [103,104]. Third, recent studies have identified a universal phenomenon in which single-cell behavior strongly affects the overall population performance [84,85,86,89]. These observations have positioned TF-based biosensors as essential tools for studying single-cell metabolism [105,106]. The advantages of TF-based biosensors lie in their ability to provide information on the real-time metabolite dynamics of living cells with high specificity as well as their ability to scale to thousands or even millions of cells when coupled with high-throughput microscopy or FACS [107]. Furthermore, extensive evidence has shown that the performance of engineered microbes can be affected by gene expression burden and stress from engineering [108,109,110]. New biosensors that can detect such burden and stress will be useful as diagnostic tools and for alleviating their effects on cell performance.

While current knowledge and strategies have made significant advances in engineering metabolite biosensors, the complexity of microbial host metabolisms and regulation may still cause deviations in a sensor’s performance. As the general understanding of sensor-host interactions and model-based tools continue to develop [111], there is hope that metabolite biosensors can be engineered more accurately and efficiently to support a wide range of biotechnological applications.

## Figures and Tables

**Table 1:** Advantages and disadvantages of tuning strategies for biosensors.

Tuning Strategy	Advantages	Disadvantages	References
Promoter engineering	Several approaches, including changing the number or location of TF operator sites and introducing point mutations to the TF operator sites or the −35 and −10 binding sites; useful for fine-tuning sensitivity, detection range, dynamic range, cooperativity, or signal output intensity	Cannot be used to adjust sensor specificity for target metabolites	[36,37,38,39,40,41]
TF engineering	Can tune binding with target metabolite or DNA; useful for fine-tuning specificity, sensitivity, and dynamic range	Requires knowledge of TF structure and binding mechanism; TF expression level changes with cell growth conditions; mutations specific to one TF (e.g., specific base substitutions in one TF ligand-binding domain) may not be readily applicable to other TFs	[36,40,41,42,43,44,45,46,47,48]
RBS engineering	Can be used to control the rate of TF or reporter protein production; can be combined with transcriptional control for multi-layered control strategies; useful for fine-tuning the dynamic range	Cannot be used to adjust sensor specificity for target metabolites	[49,50,51,52,53]

## Data Availability

No new data were created or analyzed in this study. Data sharing is not applicable to this article.
